# European madness 1910–1980: lessons for today from Alastair Morgan's *Continental Philosophy of Psychiatry: The Lure of Madness*

**DOI:** 10.1192/bjb.2023.79

**Published:** 2024-12

**Authors:** George Ikkos

**Affiliations:** Department of Psychiatry, Royal National Orthopaedic Hospital, London, UK

**Keywords:** Schizophrenia, madness, critical phenomenology, mental health activism, mad studies

## Abstract

In *Continental Philosophy of Psychiatry: The Lure of Madness* Alastair Morgan surveys the contributions of a loosely conceived school of psychiatrists, philosophers and social theorists to understanding and responding to madness during the years 1910–1980. Taking my cue from him, I highlight some of the contributors discussed in Morgan's book and reflect that although madness may be difficult or even impossible to articulate effectively in discourse it remains a ‘limit experience’ which demarcates and illuminates the contours of other thinking and being, including reason and activism. I discuss social and cultural factors that have dulled clinicians’ sensitivities to the sounds of madness in recent decades and advocate the need for a reappraisal of our expertise and for a new activism today. What may at first appear as a failed clinical-philosophical tradition remains of professional relevance in today's rapidly transforming circumstances of practice both as inspiration and as cautionary tale.

‘… is there any witnessing to madness? Who can witness? Does witnessing mean seeing? Does it have an object? Is there any object? Is there a possible third, that might provide a reason without objectifying?’

Jacques Derrida (quoted by Morgan: p. 279)^1^

Alastair Morgan is senior lecturer in mental health nursing at the University of Manchester and identifies as a critic of psychiatry. If so, his critique is sensitive, even sympathetic. The central aim of his book *Continental Philosophy of Psychiatry: The Lure of Madness* is the ‘rescue of a minor psychiatry that was open to difference and plural in its practice and outlook. Such a rescue is an attention to parts of history that have been passed by, neglected or overlooked’ (p. viii).^1^ This, he hopes, will offer an image of psychiatry that is ‘pluralistic, creative, and rebellious’ (p. 8). In what follows, I offer a personal reflection in response, not a review. I am grateful to Dr Morgan and his fascinating book for helping bring out some ideas in me about Western political economy and culture, and the culture of psychiatry within it, that I have carried for some time inside but had not found the words to articulate.

The period examined is 1910–1980 and the volume is divided into five parts, entitled respectively ‘Three inclusive exclusions’, ‘Through a glass darkly’, ‘It's a mad world’, ‘A certain madness must watch over thinking’ and ‘Antipsychiatry and madness’. The very first sentences read: ‘This book begins with an experience of philosophical failure. The tradition of continental philosophy of psychiatry attempted to understand madness using the methodological principles of the newly formed human sciences: understanding, empathy and the reconstruction of narrative meaning. However, when faced with madness, these methodologies crumbled’ (p. 1).^1^ Indeed!

## Clinical practice, thinking and activism

Here I focus only on some of the contributors to the discourse and practices that Morgan surveys and divide these into clinicians, thinkers and activists. This is my division, not his. I write as a clinician. However, some abstract concepts are referred to and include self, alienation, freedom, reason, unreason, etc. I conclude by discussing social and cultural factors that have dulled clinicians’ sensitivity to the sounds of madness in recent decades and suggest the need for a reappraisal of our expertise. I advocate the need for a new clinical sensitivity and professional activism in today's changed circumstances.

### Clinicians

Morgan helpfully begins and concludes his volume with the work of psychiatrists. The first is psychiatrist-turned-philosopher Karl Jaspers’ *General Psychopathology* (1911). Other early thinker-practitioners discussed include Eugen Bleuler, Carl Jung and Eugène Minkowski. Key later psychiatrists include R.D. Laing and David Cooper. Interestingly, if Morgan nourishes any hostility towards psychiatry, this pales by comparison with that experienced by Bleuler at the hands of German psychiatrists because of his sympathetic response to Sigmund Freud's ideas (Ch. 3) (here and below, chapter numbers refer to *Continental Philosophy of Psychiatry*).^1^

Morgan is a philosopher with a doctorate on the theme ‘Life does not live’, the epigraph to Frankfurt critical theorist T.W. Adorno's (1903–1969) glum aphoristic masterpiece *Minima Moralia*.^2^ In the light of such preparation, it is not surprising that he has a lot to say about theories that view schizophrenia as alienation and withdrawal from life itself, a disorder of ‘pre-reflective experience [ … ] such as lived time, vitality, embodied presence, minimal self (ipseity) and an immersed presence in the world’ (p. 67).^1^ This discourse, though insufficiently familiar to many psychiatrists today, remains of relevance to our contemporary phenomenological psychopathology;^3^ and not only in the work of Josef Parnas^4^ (https://easenet.dk/) and Louis Sass.^5^ No failure here! The discussion is illuminating, and it is interesting to read how ‘Freud: the reluctant philosopher’^6^ stands shoulder to shoulder with others more committed to this discipline when discussing paranoid psychosis (Ch. 4).

A word of caution. Philosophical formation is no guarantee of clinical success. Of interest is the case of Ellen West, a patient treated by renowned Swiss psychiatrist Ludwig Binswanger (1881–1966) (Ch. 8). It caused a storm of controversy after he published regarding his anguished decision to allow her to leave his clinic at a time when his ‘existential analysis’ had led him to conclude that suicide was inevitable, which is indeed what happened soon after her discharge from Bellevue Hospital in Kreuzlingen. As Morgan points out, today we would see that she had been crushed repeatedly as a victim of patriarchal misogynistic oppression, something not addressed by Binswanger. My reading of Morgan's retelling also suggests that West's was a reasonably straightforward case of treatable major depression diagnosable on grounds of descriptive psychopathology.^7^ This, of course, does not entail dismissal of ‘existential analysis’, only recognition of its limitations and the need to complement it by other means. Such complementation, I would suggest, may not simply consist of psychiatric treatments [such] as medication and psychotherapy, but include socially critical political commitments against patriarchy and misogyny, racism, etc. Because psychiatrists have often been followers rather than leaders in such matters,^8–10^ I turn now to some innovative thinkers discussed by Morgan.

### Thinkers

A look at the index shows that Michel Foucault's (1926–1984) name comes second only to Freud's in number of references, with Martin Heidegger's third. A welcome strength of the volume is that we can trace some of the evolution in his thinking across parts II–VI. Part IV, ‘A certain madness must watch over thinking’, is about his (Ch. 13 and Ch. 14) and psychiatrist and psychoanalyst Jacques Lacan's (1901–1981) (Ch. 15) theories of madness. Morgan explains in detail how for Lacan ‘Madness, however disavowed, lies at the heart of reason’ (p. 250),^1^ a credible proposition to my mind, even if others may differ. It is echoed in the important Foucault–Jacques Derrida (1930–2004) philosophical debate on madness.

Morgan explores the Foucault–Derrida debate. He warns that it ‘consists of a series of texts that concern a wide array of philosophical questions in an often confused and confusing manner. The term “debate” is a misnomer as the first response to Derrida's critique was a lack of contact between the two philosophers for ten years following Derrida's initial lecture’ (p. 258). The key issue was Foucault's formulation and use of the philosophically abstract concepts of reason, unreason and madness, particularly his insistence in early editions of his *History of Madness*^11^ that reason silenced unreason and madness during the Enlightenment. Much of the debate centred around Descartes’ *Discourse on Method*^12^ as the harbinger of the Enlightenment, including the great rationalist's reference to madness in that text. Derrida suggested that there is ‘a paranoiac grandiosity’ (p. 264)^1^ in Descartes’ ‘solipsistic statement’ that ‘I think, therefore I am’.

‘The [Enlightenment's] moment of philosophical certainty and the grounding for a philosophical system do not rest upon the exclusion of madness but rather on a strange madness within philosophy’ comments Morgan (p. 264), arguing that Foucault and Derrida came to echo Lacan in that ‘ultimately [madness itself] cannot be spoken about or speak, but that it nevertheless registers; it haunts reason and watches over reason from a distance’ (p. 250). Among the consequences that followed for Foucault, writes Morgan, one was to allow explicitly for the investigation of mental illness empirically in our contemporary scientific way, quite apart from any abstract speculations about ‘reason’ and ‘unreason’. Another was to shift his focus from the relation between reason, unreason and madness to that between transgression and madness, hence the title of Ch. 14 and subtitle of the book *The Lure of Madness*. Morgan concludes ‘At his best, Foucault traces the negative space of madness as both limit and critique of a certain fate of Western reason and responds to the suffering of madness. At his worst, he outlines a concept of madness as unreason that lies in an empty transgression of reason’ (p. 281). What interested Foucault consistently, however, in his mature writing was how power shapes institutions, thinking and practices across history, including psychiatry's history. I move on now therefore to two psychiatrist-activists who took power head on.

### Activists

Frantz Fanon (1925–1961) ‘is the most complete example of the philosopher-psychiatrist’ avers Morgan (p. 224).^1^ In his thesis on Friedrich's ataxia, where he examined issues of freedom in neurological illness, Fanon combined phenomenological, gestalt and Lacanian approaches. He argued that ‘There is a pathology in the heart of freedom, in that the more one asserts an empty autonomous freedom, the more this freedom dissolves into anonymity’ (p. 227). The value of Fanon's work according to Morgan arises from the fact that it ‘begins with an understanding that the “mad person” is one who is “foreign to society” and therefore all psychiatric work is an attempt at disalienation, an effort to return the person to a place in society [ … ] For Fanon, the attempt at disalienation that led him to psychiatry as a profession demands radical questioning of wider pathologies in society [ … ] Fanon returns to the central phenomenological question but with a critical lens’ (p. 224). A turning point for him was when he was travelling by train and a child turned to its mother and said ‘Look, a Negro!’. Fanon summarised ‘I came into the world imbued with the desire to attain to the source of the world and then I found that I was an object in the midst of other objects’ (quoted by Morgan, p. 231). His first major work, *Black Skin, White Masks*, a pioneering treatise on what has become the study of pathologies of recognition, is a ‘radical melange of phenomenology, psychoanalysis, literary criticism and political rhetoric’ (p. 226). Importantly, ‘Rather than madness as a breakthrough, Fanon consistently views it as a lack of freedom and that the psychiatrist's role is to try and restore a sense of agency and freedom to the person’ (p. 227).

Issues of mental health and illness and their relation to freedom have continued to be discussed since.^13^ What Morgan's account suggests is that for Fanon freedom is not an abstraction, but an experience rooted in one's body and circumstances and it always needs to be examined with explicit reference to their concreteness. Furthermore, substantive freedom emerges from lively engagements with these, not their empty transgression. Consistent with this, Morgan places Fanon's ideas and practice in the context of his biography and thus helps deepen understanding of their nature and significance.^14^ Fanon was born in 1925 in Fort-de-France, in the wretched conditions of French colonial Martinique in the Caribbean. In 1944, he joined General Charles de Gaulle's (1890–1970) ‘Free French’ and fought in Morocco, Algeria and, finally, in France, where he was appalled by the racism with which the African Senegalese were treated, even worse than the Caribbeans. Following the war he trained in medicine in Lyon and psychiatry in the Saint-Alban hospital, which had previously distinguished itself by sheltering resistance fighters and by protecting its patients from starvation in ways that other French mental hospitals had not during General Pétain's pro-Nazi Régime de Vichy (1940–1944). By 1952–1953, when he joined the staff, it had also pioneered the ‘institutional psychotherapy’ approach to treatment, a distinctly French approach to group and community therapy influenced by Lacan and Marxism. He took his ideas to Algeria, where he first challenged colonial psychiatrists’ racist theories of ‘the North African syndrome’ and later joined the Algerian Front de Libération Nationale (FLN), endorsing its violent insurrection against France's brutal colonial occupation. In Algeria he sheltered local insurgents in his hospital in Blida and then, in exile in Tunis, he combined anti-imperialist political leadership with psychiatric practice with patients traumatised by the French reaction.

A word of caution about Fanon too. He is clearly a kind of hero for Alastair Morgan and his reputation has been further enhanced in our contemporary context of ‘Black lives matter’. It is a tribute therefore to the author's intellectual and ethical integrity that he does not shy away from referring to Fanon's and his Saint-Alban colleagues’ engagement in the ghastly and aptly named ‘annihilation therapy’. This combined electroconvulsive therapy, insulin therapy and sleep therapy in the context of the institutional psychotherapy in this hospital. Morgan quotes (p. 230) from Fanon's and François Tosquelles’ paper ‘On some cases treated with the Bini method’: ‘The point here is to situate annihilation therapy through repeated shocks within an institutional therapeutic performance’. Morgan continues incredulously: ‘The point of the shock treatment was not an end in itself but to try and create a kind of “new birth” in the patient, although quite what was left after Fanon and François Tosquelles had finished beggars belief, as they describe the following course of treatment with one patient: “Five days of annihilation treatment, she had seventeen electroshocks … forty sessions of insulin shock therapy”’ (p. 230). Fanon thus emerges as a somewhat ambiguous figure rather than an undisputed hero. Also, his endorsement of political violence has divided opinion. Some demure from it, others have criticised him heavily, yet others, including existentialist philosopher Jean-Paul Sartre, joined him in advocacy for the FLN.

Another psychiatrist noted by Morgan who made direct use of experiences of oppression and war to challenge psychiatric institutions and society was Franco Basaglia (1924–1980). He had taken part in resistance to Benito Mussolini's (1883–1945) fascist dictatorship (1922–1943) and had been imprisoned in Italy.^15,16^ An existential phenomenological psychiatrist influenced by Sartre, he abandoned his academic post in Padua after the war to work in Italy's primitive and despised asylum system in Gorizia and Trieste on the north-east coast of the Adriatic Sea. Noting the similarities between mental hospitals and prisons, even the smells, he set out to close the asylums both in the region and nationally. His ‘Psichiatria Democrática’ movement was radically political and succeeded in the Italian parliament with Law 180, which mandated the closure of all mental hospitals from 1978.

## Discussion

*Continental Philosophy of Psychiatry* begins with Jaspers’ *General Psychopathology* and ends in 1980, the year Basaglia died. The three decades following the Second World War (‘*les trente glorieuses*’, 1945–1975) were characterised by optimism regarding social reform and inclusion, and the 1960s and 1970s by de-institutionalisation and diversity of thinking in psychiatry. Then the election of Margaret Thatcher in 1979 (and the triumph of neoliberalism that followed), the award of the Nobel Prize for Medicine to the inventors of the brain CAT scan the same year (and the ‘Decade of the Brain’ in the 1990s) and the publication also in 1979 of Jean-François Lyotard's *La Condition Postmoderne: Rapport sur Le Savoir* (*The Postmodern Condition: A Report on Knowledge*)^17^ signalled great changes in culture, society and medicine, the consequences of which we are still living with in psychiatry today.

Morgan's epilogue is consistent with assessments of the impact of the above changes.^18^ The political economic imperatives of neoliberalism, combined with advances in technology in medicine and the growth of information technology, as well as the shortcomings of the continental philosophy of psychiatry that Morgan exposes, banished references to madness in psychiatric research, training and practice. Whether in the DSM, epidemiology, genetics, physiology or brain imaging, certainty was sought in aggregate numbers^19^ at the expense of discourse, narrative and attention to difference. This suited the emerging managerialism in health services too. Meanwhile, neoliberal consumerism in health fuelled a welcome and growing service user movement and its advocates. A significant, though not the only, perspective that has gained some strength as a part of the movement has been normalisation and denial of mental illness.^20^ Thus, insights from the continental tradition into both the reality and difference of madness and the limitations of ‘understanding, empathy and the reconstruction of narrative meaning’ (p. 1)^1^ were glossed over in public debate too. Elsewhere, Morgan has explicitly criticised such views, focusing specifically on the ‘power threat meaning framework’ (PTMF).^21^

Since the turn of the century, we have experienced the neoliberal financial crisis of 2008, the COVID-19 pandemic, even the Russian invasion of Ukraine. These, together with the emergence of social media and the failure of the ‘Decade of the Brain’ to deliver its promised clinical outcomes, have fundamentally changed the social and cultural landscape and created new opportunities and forums for communication among diverse lay and professional groups and renewed challenges to clinical authority. However inarticulate the voice of madness may be or difficult to make sense of, we must find a space for it to mumble, whisper or howl – give it voice, so to speak.^22,23^ And we must be wise to its haunting and join our own voice with it in protest when needed.

Without forsaking our expertise, we need to reconsider its nature and valence.^24,25^ This requires engaging in meaningful ways in ongoing dialogue with the service user movement about our respective identities and ways of doing things and mutual expectations.^26^ One practical way to begin immediately would be through proper attention to ‘mad studies’ at all stages of training and continuing professional development of psychiatrists.^27^ We also need to radicalise our political involvement with service user organisations, at both national and local level.^28^ Neoliberalism has failed our patients.^29,30^ Now that debate is underway on whether we are moving from neoliberalism to neo- or technofeudalism^31–33^ there will likely be a need for further radicalisation in mental health, somewhat like Fanon and Basaglia although not identical.

In the light of Morgan's instructive volume, some words of caution are imperative too. Alluring though the idealisation of madness may be, it has run its course. Even though Morgan flirts with this allure, it is no breakthrough. Just perhaps it may be some kind of evolutionary adaptation.^34,35^ And necessary though philosophical and ideological discourse might be for a fuller understanding, they have pitfalls of their own. Although it may have fallen short in terms of fulfilling expectations, empirical and biological research in psychiatry has an essential place in dialogue and activism too. A vast array of data has accumulated through painstaking work and no complex society can hope to address issues of mental health and illness without reference to it, although not in a hegemonic but an irrevocably pluralist environment.^36,37^

## Conclusion

Morgan ends by quoting Adorno: ‘I would maintain that Wittgenstein's statement that “what we cannot speak of we must pass over in silence” is the anti-philosophical statement par excellence. We should insist instead that philosophy consist in the effort to say what cannot be said’ (p. 413). I would add that psychiatry should be one way of understanding and caring for that part of it we all call madness, sometimes psychosis too. To do so we must approach madness from a position of respect for difference, even its incomprehensibility,^38^ and listen! And speak up. Speak truth to power.

## Data Availability

Data availability is not applicable to this article as no new data were created or analysed in this study.

## About the author

**George Ikkos** is a consultant liaison psychiatrist in the Department of Psychiatry at the Royal National Orthopaedic Hospital, London, immediate past Chair of the Royal College of Psychiatrists’ History of Psychiatry Special Interest Group (HoPSIG) and former President of the Royal Society of Medicine's Pain Medicine and Psychiatry Sections. He is joint editor of *Mind, State and Society: Social History of Psychiatry and Mental Health in Britain 1960–2010*, which was runner up (silver) in the Association of American Publishers’ PROSE 2022 Awards, in the section History of Science, Medicine and Technology (available open access from https://doi.org/10.1017/9781911623793).
